# Implications of NLRP3 Suppression Using Glibenclamide and miR-223 against Colorectal Cancer

**DOI:** 10.3390/ph17030299

**Published:** 2024-02-26

**Authors:** Shaimaa Hamza, Ekaterina E. Garanina, Layaly Shkair, Mohammad Alsaadi, Svetlana F. Khaiboullina, Gulcin Tezcan

**Affiliations:** 1 Institute of Fundamental Medicine and Biology, Kazan Federal University, 420008 Kazan, Russiaekaterinaakagaranina@gmail.com (E.E.G.); shkair.layaly.94@gmail.com (L.S.); mhmad.alsadi19955@hotmail.com (M.A.); 2 Department of Fundamental Sciences, Faculty of Dentistry, Bursa Uludag University, Bursa 16059, Turkey

**Keywords:** NLRP3, inflammasome, glibenclamide, microRNA-223, colorectal cancer, metastasis

## Abstract

The NLR family pyrin domain containing 3 (NLRP3) promotes the growth of colorectal cancer (CRC). However, the therapeutic effect of NLRP3 inhibition on CRC cell progression is controversial. This study comparatively investigated the therapeutic effect of a pharmacological NLRP3 inhibitor, glibenclamide (gli), and the post-translational suppression of NLRP3 by miR-223 on CRC cell progression in HCT-116 and HCT-15 cells. LPS and ATP were used to activate Gli-treated and LSB-hsa-miR-223-3p (WT^miR-223^)-expressing HCT-116 cells. NLRP3.AB.pCCL.sin.cPPT.U6.miR-223-Decoy.hPGK.GFP.WPRE plasmid (D^miR-223^) was the negative control for miR-223 expression. NLRP3, gasdermin D, and BAX expressions were analyzed using western blotting. Real-time PCR detected the RNA expression of autophagy-related genes ATG5, BECN1, and miR-223 in non-transfected cells. ELISA analyzed IL-1β and IL-18 in the medium. MTS-1, annexin V, wound-healing, and sphere-invasion assays were used to assess cell viability and progression. A multiplex cytokine assay detected proinflammatory cytokine secretion. LPS–ATP-activated NLRP3 produced gasdermin D cleavage, released IL-1b and IL-18, and activated cell migration and sphere invasion. In contrast, reduced cell growth, miR-223 expression, IFN-γ, CXCL10, and LIF secretion were found in cells after inflammasome activation. Both gli and WT^miR-223^ induced autophagy genes ATG5 and BECN1 and reduced the NLRP3 activation and its downstream proteins. However, while gli had a limited effect on the production of IFN-γ, CXCL10, and LIF, WTmiR-223 increased the release of those cytokines. In addition, gli did not suppress cell growth, while WTmiR-223 promoted apoptosis. Notably, neither gli nor WTmiR-223 effectively prevented sphere invasion. These data suggest that, while WT^miR-223^ could have a better anticancer effect in CRC compared to gli, the sole usage of miR-223-mediated NLRP3 suppression may not be sufficient to prevent CRC metastasis.

## 1. Introduction

Colorectal cancer (CRC) is the third most common cancer worldwide [1]. One of the main risk factors of CRC tumorigenesis is inflammation [2,3]. NLR family pyrin domain containing 3 (NLRP3) plays a central role in the pathogenesis of CRC inflammation [4].

High levels of NLRP3 have been detected in CRC tumor tissues with advanced tumor, node, and metastasis (TNM) stages, which are characterized by distant metastasis, vascular invasion, and positive lymph nodes [5]. NLRP3-mediated proinflammatory cytokine secretion triggers the metastasis of CRC cells through epithelial–mesenchymal transition (EMT) [6,7,8]. Therefore, inhibiting NLRP3 was suggested to suppress tumor aggression by reducing the release of inflammatory cytokines [5].

Based on the role of the inflammasome in the pathogenesis of cancer, therapeutic approaches aimed to inhibit the NLRP3-induced proinflammatory cytokines were tested [9]. Recently, an IL-1β receptor antagonist, anakinra, administered alone was found to be insufficient in reducing tumor size in cancers, including CRC [10,11]. In addition to IL-1β receptor antagonists, the function of NLRP3 could be suppressed by inactivating caspase-1, blocking the formation of the inflammasome complex, or inhibiting the upstream signals [5,12]. Our previous studies revealed that VX765, a caspase-1 inhibitor, increases the secretion of pro-angiogenic cytokines from cancer cells [13]. However, the effect of preventing NLRP3-induced proinflammatory cytokine secretion by inhibiting the upstream signals and blocking the formation of the inflammasome complex assembly on cancer cell growth remains unknown [5,12].

Glibenclamide (gli) blocks the assembling signal for NLRP3 activation by inhibiting the P2X7, a purinoceptor for ATP [14]. While several studies have reported the antiproliferative effect of gli in several cancer cell lines [15,16,17], there are also data suggesting that gli increases the risk of cancer in diabetes, a disease in which inflammation is one of the risk factors [18,19]. It should be noted that the effect of gli on CRC remains unknown.

Suppression of NLRP3 mRNA translation using microRNA (miRNA) could block the formation of the inflammasome complex [20,21,22]. miRNA is a noncoding RNA molecule with a length of 9–24 nucleotides that modulates the stability and translation of a target mRNA by complementarily binding to its untranslated region (UTR) [20,21]. miR-223 has an evolutionary conserved binding site at the 3′UTR of the NLRP3 transcript [22]. Although miR-223 has been implicated in reducing NLRP3 expression and IL-1β secretion in a variety of inflammatory diseases [23], data on its effects in CRC are inconsistent [24,25,26]. Therefore, in this study, we investigated the effect of the P2X7 inhibitor, gli, as a blocking agent for assembling NLRP3 and the effect of miR-223 as a posttranslational inhibitor of NLRP3 in HCT-116 and HCT-15 CRC cell lines under NLRP3 activation.

## 2. Results

### 2.1. NLRP3 Activation Suppresses Cell Growth through Proinflammatory Cytokine Secretion, While gli Fails to Oppose the Inflammasome’s Effects

**Figure 1:**
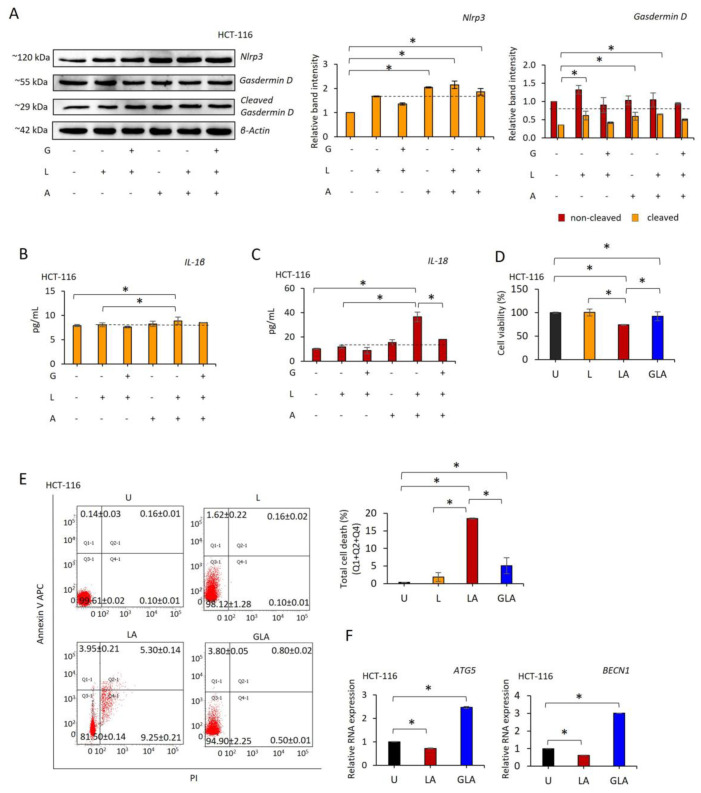
Effect of gli on NLRP3 function in HCT-116 cells. LPS (1 µg/mL, 3 h) was used to prime NLRP3, followed by ATP (5 mM ATP; 25 min) to activate the inflammasome. Gli (50 ug/mL) was added 24 h before NLRP3 induction to inhibit the inflammasome. (**A**) Western blot of NLRP3 and gasdermin D proteins. (**B**) ELISA assesses IL-1β and (**C**) IL-18 secretion. (**D**) The MTS assay values of treated cells were normalized to the untreated sample. (**E**) The Annexin V assay of cell viability. (**F**) ATG5 and BECN1 RNA expression levels. Data represent three biological replicates, and *p*-value was calculated using the one-way ANOVA model with Tukey’s post hoc tests. * *p* < 0.05. U: Untreated, L: LPS only, LA: LPS–ATP, GLA: Gli–LPS–ATP.

LPS–ATP (*p* = 0.001) increased RNA expression of NLRP3 in HCT-116 (Figure S1A) and HCT-15 (Figure S1A) cells compared to untreated cells. Consistently, ATP-only (*p* = 0.003) and a combination of LPS–ATP (*p* = 0.001) increased NLRP3 protein expression in HT116 cells compared to untreated cells (Figure 1A). Also, the cleavage rate of gasdermin D increased in LPS-only (*p* = 0.009), ATP-only (*p* = 0.018), and LPS–ATP (*p* = 0.003) cells compared to untreated HCT-116 cells (Figure 1A). In contrast, although gli reduced the LPS–ATP-induced NLRP3 level, it did not reach the level in the untreated cells (*p* = 0.039). Additionally, gli did not affect the level of LPS–ATP-induced gasdermin D cleavage.

The secretion of interleukin (IL-1β) (*p* = 0.001) and IL-18 (*p* < 0.001) was increased in LPS–ATP-treated compared to the untreated HCT-116 cells. Interestingly, LPS-only treatment did not affect cytokine secretion (Figure 1B,C). Conversely, pretreatment of HCT-116 cells with gli reduced the release of IL-18 compared to LPS–ATP-treated (*p* < 0.001). It should be noted that IL-1β was not affected by gli compared to LPS–ATP (Figure 1B,C).

The MTS assay showed that gli concentrations between 20 and 500 µg/mL failed to inhibit proliferation in HCT-116 and HCT-15 cells (Figure S1C,D). In addition, LPS-only and gli–LPS–ATP did not affect HCT-116 (Figure 1D) and HCT-15 (Figure S1E) cell growth compared to untreated cells. In contrast, LPS–ATP substantially decreased the cell proliferation rate (*p* < 0.001, Figure 1D and Figure S1E). Consistently, the total cell death rate was increased by LPS–ATP compared to untreated and LPS-only treated HCT-116 and HCT-15 cells (*p* < 0.001, Figure 1E and Figure S1F). Similarly, gli inhibited the cell-killing effect of LPS–ATP (Figure 1E and Figure S1F). Furthermore, ATG5 (*p* < 0.0001) and BECN1 (*p* < 0.0001) RNA expressions were increased by gli, while LPS–ATP reduced ATG5 expressions in HCT-116 (*p* < 0.0001, Figure 1F) and BECN1 in HCT-116 and HCT-15 cells (*p* < 0.0001, Figure 1F and Figure S1G).

These data demonstrate that LPS-priming of NLRP3 does not affect CRC cell growth. However, the second stimulus by ATP reduces cell growth and leads to proinflammatory cytokine secretion, possibly due to increased gasdermin D cleavage. In contrast, gli–LPS–ATP reduces NLRP3-mediated cell inhibition and proinflammatory cytokine secretion. The cell-protective effect of gli could be autophagy-dependent because it induced RNA expression of genes ATG5 and BECN1 without leading to apoptosis.

### 2.2. Gli-Induced CRC Cell Invasion

**Figure 2:**
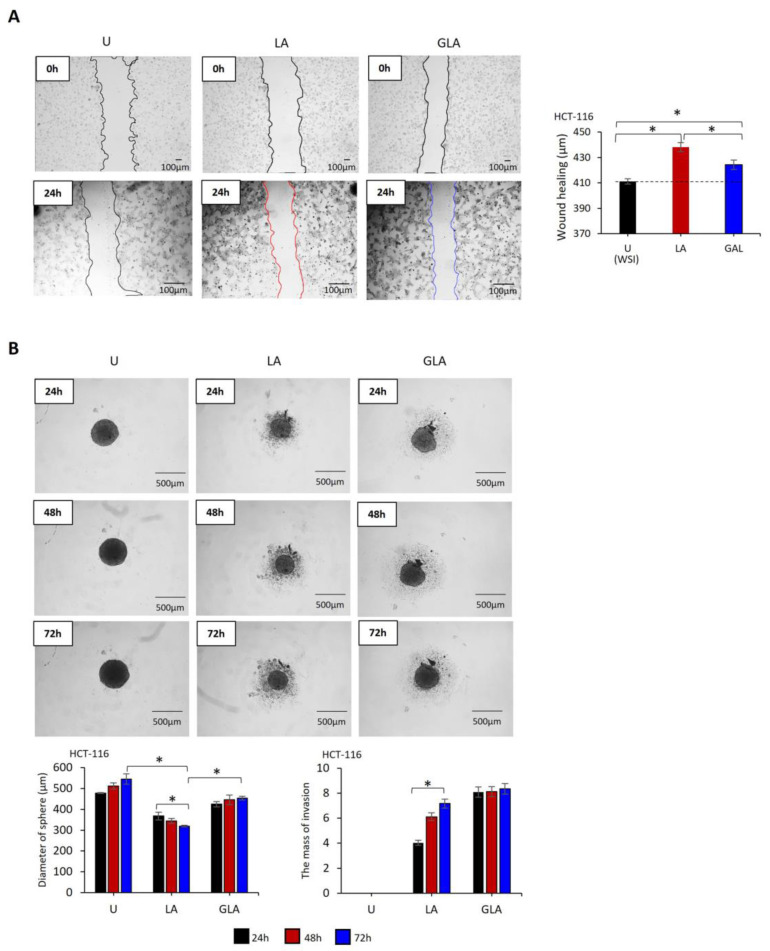
The effect of gli inhibition of NLRP3 on migration and sphere formation of HCT-116 cells. LPS (1 µg/mL, 3 h) was used to prime NLRP3, followed by ATP (5 mM ATP; 25 min) to activate the inflammasome. Gli (50 ug/mL) inhibited NLRP3 24 h before LPR–ATP induction. (**A**) Wound-healing of HCT-116 cells. Images were captured before LPS treatment (time 0) and 24 h after ATP treatment. (**B**) Sphere formation and invading capacity of HCT-116 cells. Images were captured 24–72 h after ATP treatment. Images were analyzed using Image J 1.53s software (NIH, Bethesda, MD, USA). Data represent three biological replicates. The *p*-value was calculated using an independent sample *t*-test. * *p* < 0.05 U: Untreated, LA: LPS–ATP, GLA: Gli–LPS–ATP, WSI: Wound size at the initiation.

The wound-healing rate in LPS–ATP-treated cells was higher than that of untreated cells at 24 h (HCT-116: t = 11.98, *p* < 0.001, Figure 2A; HCT-15: t = 4.19, *p* = 0.014, Figure S2A). In contrast, gli–LPS–ATP decreased wound healing compared to LPS–ATP-treated cells (HCT-116: t = −5.57, *p* = 0.001, Figure 2A; HCT-15: t = 2.05, *p* = 0.132, Figure S2A). However, despite the presence of gli, the wound healing induced by LPS + ATP did not reach the same level in untreated cells (Figure 2A and Figure S2A).

Untreated and LPS–ATP or gli–LPS–ATP-treated cells formed spheres within 24 h. The size of the spheres formed by LPS–ATP-treated (HCT-116: t = −14.9, *p* < 0.001; HCT-15: t = −6.2, *p* = 0.003) and gli–LPS–ATP-treated cells (HCT-116: t = −10.2, *p* = 0.001; HCT-15: t = −3.8, *p* = 0.018) was smaller than that of the untreated cells at 72 h. Additionally, LPS–ATP promoted the invasion of sphere-detached cells, as evidenced by the distribution of cells around the sphere, indicating active migration of cells from the sphere. The invasion of sphere-detached cells increased in LPS–ATP-treated cells over 72 h (HCT-116: t = 9.7, *p* = 0.001; HCT-15: t = 11.9, *p* < 0.001). In addition, the sizes of the spheres formed by gli–LPS–ATP-treated cells was larger than those formed by LPS–ATP (HCT-116: t = 9.5; *p* = 0.005, Figure 2B) (HCT-15: t = 4.59, *p* = 0.010, Figure S2B). Although the mass of sphere-detached cell invasion around the gli–LPS–ATP-treated spheres was not affected over time, it was larger than the mass of sphere-detached cell invasion around LPS–ATP-treated spheres after 72 h (HCT-116: t = 6.2, *p* = 0.003; HCT-15: t = 13.8, *p* < 0.001, Figure S2B).

### 2.3. NLRP3 Has a Dual Effect on Proinflammatory Cytokine Release in HCT-116 Cells, While gli Promotes Angiogenesis

**Figure 3:**
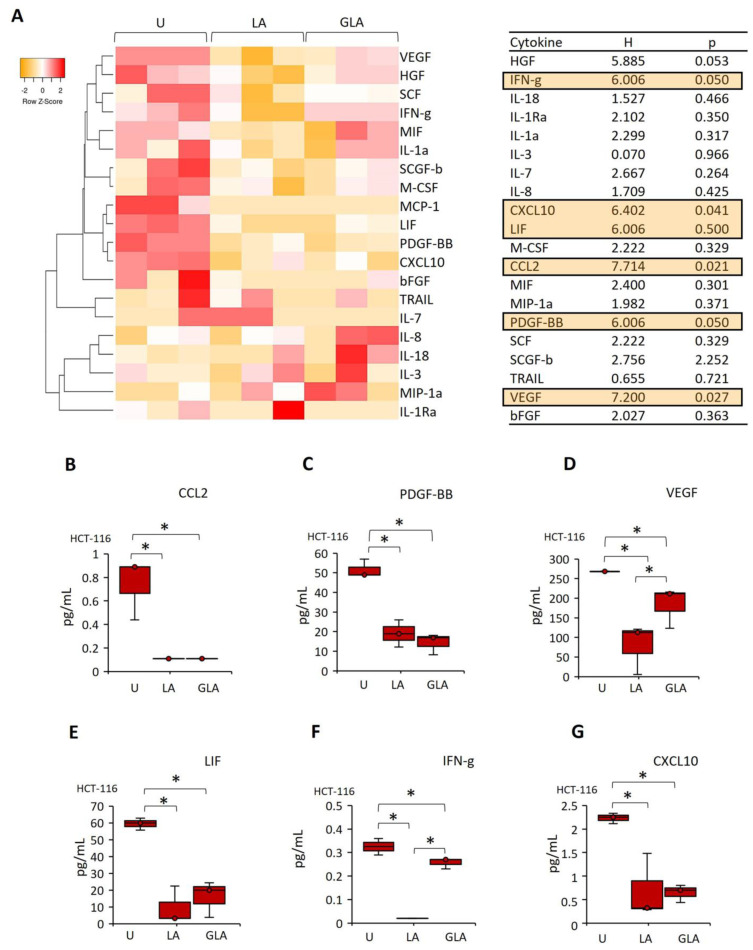
The effect of gli on cytokine secretion in LPS–ATP-induced HCT-116 cells. (**A**) The heat map shows changes in cytokine secretion patterns in HCT-116 cells after LPS–ATP and gli–LPS–ATP treatments. Cytokines with concentrations below the detection level were excluded from the heatmap graphs. (**B–G**) The cytokines, which were affected by LPS–ATP and gli–LPS–ATP. Data represent three biological replicates. The *p*-value was calculated using a Kruskal–Wallis test. H: Test statistic for the Kruskal–Wallis test. * *p* < 0.05 U: Untreated, LA: LPS–ATP, GLA: Gli–LPS–ATP.

The cell culture medium levels of 48 cytokines were analyzed 24 h after LPS–ATP and gli–LPS–ATP treatment of HT116 cells (Figure 3A). Secretion levels of chemokine ligand 2 (CCL2), platelet-derived growth factor-BB (PDGF-BB), vascular endothelial growth factor (VEGF), leukemia inhibitory factor (LIF), interferon-gamma (IFN-γ) and C-X-C motif chemokine ligand 10 (CXCL10) were significantly lower in LPS–ATP- and gli–LPS–ATP-treated compared to untreated HCT-116 cells (Figure 3B–E). In contrast, gli–LPS–ATP increased the secretion of VEGF and IFN-γ in HCT-116 compared to LPS–ATP (Figure 3B,E). Gli–LPS–ATP did not affect the secretion of CCL2, PDGF-BB, LIF, and CXCL10 compared to LPS–ATP.

These data suggest that LPS–ATP promotes HCT-116 cell sphere formation and inhibits LIF secretion, which could mediate CSC maintenance [27]. In contrast, although suppressing NLRP3 using gli could induce the secretion of IFN-γ, the secretion of this cytokine is lower compared to that in untreated cells. Additionally, gli-induced VEGF secretion leads to angiogenesis [28].

### 2.4. NLRP3 Activation Suppresses the Expression of miR-223 in CRC Cells, While Adding miR-223 Reduces NLRP3 Activation

**Figure 4:**
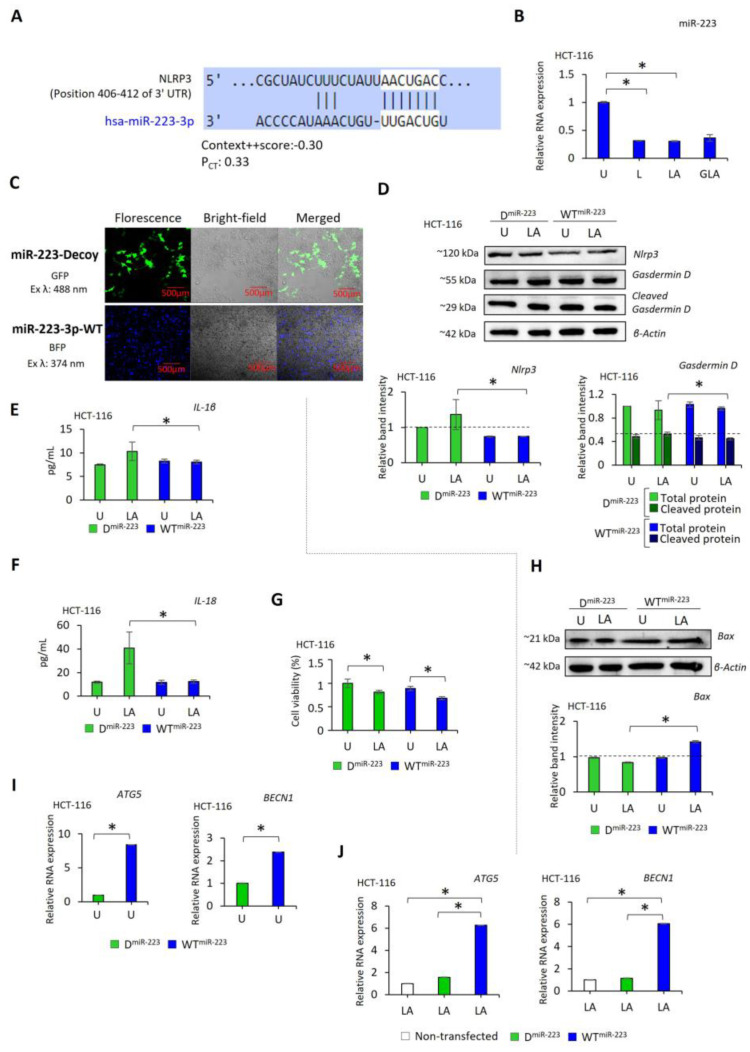
The effect of miR-223 on NLRP3 in HCT-116 cells. (**A**) The interaction of has-miR-223-3p with the 3′UTS of NLRP3 gene (Targetscan); (**B**) Effect of LPS–ATP and gli–LPS–ATP on miR-223 expression of HCT-116 cells; (**C**) Immunofluorescence (IF) analysis of the cells transfected with WT^miR-223^ (blue) and D^miR-223^ (green). WT^miR-223^ expressed cells were selected through puromycin treatment, while D^miR-223^ expressed cells were selected with flow sorting. (**D**) Western blotting of NLRP3 and gasdermin D proteins; (**E**) ELISA assessment of IL-1β; (**F**) IL-18 secretion. (**G**) The MTS assay values of D^miR-223^ and WT^miR-223^ expressing cells; (**H**) Western blotting of BAX protein. (**I**) ATG5 and BECN1 RNA expression levels in untreated and (**J**) LPS–ATP treated D^miR-223^ and WT^miR-223^ expressing cells. n = 2 for each experiment. * *p*-value was calculated using an independent sample *t*-test for (**B**) and a one-way Anova and Tukey Test for (**D**–**H**). * *p* value < 0.05. U: Untreated, LA: LPS–ATP.

A conserved interaction site for miR-223-3p is present on the 3-UTR site of NLRP3 RNA (Figure 4A) [29]. We found that the expression of miR-223 was lower in LPS-only (*p* < 0.001) and LPS–ATP-treated (*p* < 0.001) cells than in untreated HCT-116 cells (Figure 4B). Furthermore, gli–LPS–ATP did not affect the miR-223 expression compared to LPS-only and LPS–ATP in HCT-116 cells (Figure 4B). These findings suggested that LPS–ATP-mediated NLRP3 induction leads to suppression of miR-223. Additionally, gli did not restore miR-223 expression.

To clarify the role of miR-223 in NLRP3 formation, miR-223 expression of HCT-116 and HCT-15 cells was established using wild-type (WT) miR-223-3p plasmid (LSB-hsa-miR-223-3p). Also, the function of miR-223 was blocked by a decoy (D) miR-223 plasmid (AB.pCCL.sin.cPPT.U6.miR-223-Decoy.hPGK.GFP.WPRE). The HCT-116 cell transfected with LSB-hsa-miR-223-3p (WT^miR-223^) and AB.pCCL.sin.cPPT.U6.miR-223-Decoy.hPGK.GFP.WPRE (D^miR-223^) is shown in Figure 4C. WT^miR223^ caused a substantial reduction in NLRP3 RNA (Figure S3A,B) and protein compared to D^miR-223^ (*p* < 0.0001, Figure 4D). In addition, LPS–ATP significantly induced NLRP3 in D^miR-223^ compared to WT^miR-223^ cells (Figure 4D, Figure S3A,B). Additionally, while gasdermin D cleavage was similar between DmiR-223- and WTmiR-223-expressing HCT-116 cells, LPS–ATP increased the level of cleaved gasdermin D in DmiR-223 compared to WTmiR-223 cells (p = 0.006, Figure 4D). In line with this, IL-1β and IL-18 secretion were not affected by WT^miR-223^ and D^miR-223^ cells. Also, LPS–ATP did not affect the IL-1β and IL-18 secretion in WT^miR-223^ cells. In contrast, the release of IL-1β and IL-18 was enhanced in LPS–ATP-treated D^miR-223^ (*p* = 0.025 and *p* = 0.004, respectively) compared to untreated cells (Figure 4E,F). These results indicate that translational silencing of NLRP3 by miR-223 suppresses gasdermin D cleavage, as well as IL1-β and IL-18 release in WT^miR-223^ expressing HCT-116 cells. On the contrary, blocking miR-223 by D^miR223^ failed to interrupt NLRP3 activation. Additionally, LPS–ATP stimulated NLRP3 downstream proteins in D^miR-223^ expressing cells.

WT^miR-223^ did not affect the proliferation rate compared to D^miR-223^-expressing HCT-116 (Figure 4G) and HCT-15 cells (Figure S3C). However, LPS–ATP decreased the proliferation of cells expressing WT^miR-223^ (HCT-116: *p* = 0.008; HCT-15: *p* = 0.005) and D^miR-223^ (HCT-116: *p* < 0.001, HCT-15: *p* = 0.024). Additionally, WT^miR-223^ did not lead to a difference in BAX expression compared to D^miR-223^ expressing cells without stimulation (Figure 4H). However, WT^miR-223^ induced the RNA expression of ATG5 and BECN1 compared to D^miR-223^ expressing HCT-116 (*p* < 0.0001, Figure 4I) and HCT-15 cells (*p* < 0.0001, Figure S3D). Notably, LPS–ATP induced the pro-apoptotic protein BAX in WT^miR-223^, while the expression of this protein was not affected in D^miR-223^ expressing cells (*p* < 0.001, Figure 4H). In addition, LPS–ATP treatment did not affect ATG5 and BECN1 in D^miR-223^ cells, while enhancing the expression of these RNA in WT^miR-223^ cells compared to non-transfected cells (*p* < 0.0001, Figure 4J and Figure S3E). These results suggest that LPS–ATP-mediated cell suppression in WT^miR-223^ cells could be regulated by a crosstalk between autophagy and apoptosis.

### 2.5. miR-223 Decreases the Invasion of HCT-116 and HCT-15 Cells

**Figure 5:**
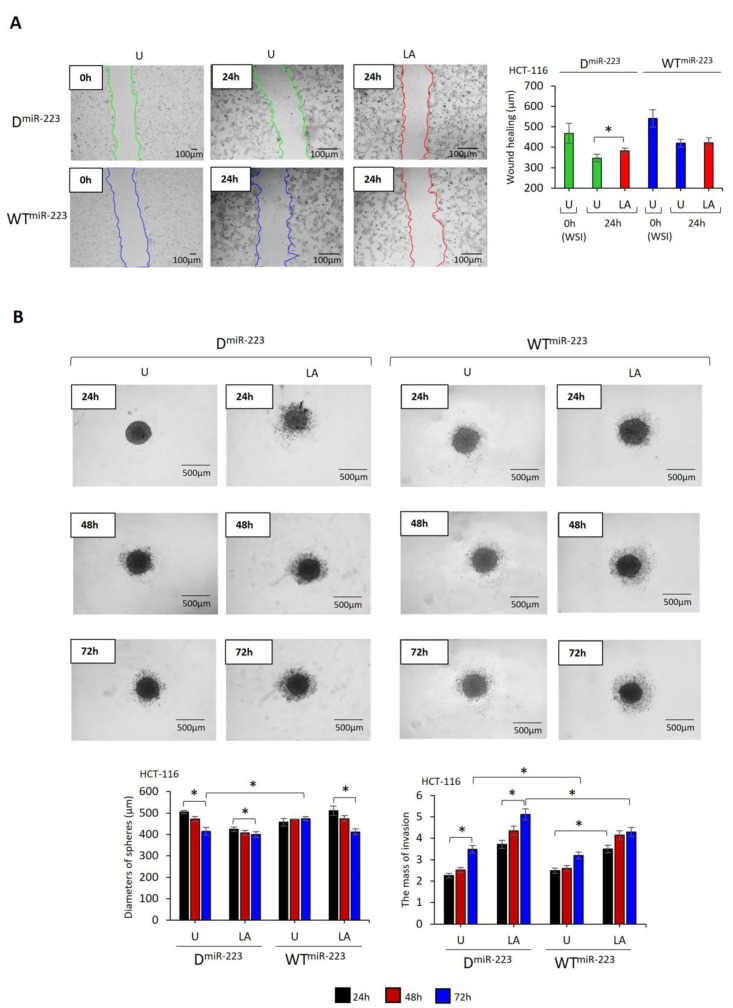
The effect of miR-223 on migration and sphere formation of HCT-116 cells. LPS (1 µg/mL, 3 h) was used to prime NLRP3, followed by ATP (5 mM ATP; 25 min) to activate the inflammasome. (**A**) Wound healing of HCT-116 cells. Images were captured before LPS treatment (time 0) and 24 h after ATP treatment. (**B**) Sphere-forming and invading capacity of HCT-116 cells. Images were captured 24–72 h after ATP treatment. Images were analyzed using Image J 1.53s software (NIH, Bethesda, MD, USA). Data represent three biological replicates. *p*-value was calculated using an independent sample *t*-test. * *p* < 0.05 D: Decoy, WT: Wild type, U: Untreated, LA: LPS–ATP, WSI: Wound size at initiation.

The wound-healing rate was significantly reduced in WT^miR-223^-expressing HCT-116 cells compared to those expressing D^miR-223^ (t = −8.8, *p* < 0.001, Figure 5A). While LPS–ATP increased wound healing in D^miR-223^ expressing cells (t = −3.117; *p* = 0.036), it was not affected in WT^miR-223^ expressing cells. In HCT-15 cells, neither WT^miR-223^ nor D^miR-223^ affected the wound healing rate. However, LPS–ATP-induced wound healing in D^miR-223^-expressing HCT-15 cells compared to untreated cells (t = 4.1, *p* = 0.014). In contrast, LPS–ATP did not affect the wound-healing rate of WT^miR-223^-expressing HCT-15 cells. In addition, after LPS–ATP treatment, wound healing was lower in WT^miR-223^-expressing HCT-15 cells compared to D^miR-223^-expressing cells (t = −3.9, *p* = 0.017, Figure S4A). These findings indicate that miR-223 either stabilizes or reduces the migratory capacity of the cells, and inflammasome activation has a limited effect on cancer cell migration.

D^miR-223^ and WT^miR-223^ HCT-116 and HCT-15 cells formed spheres within 24 h. The sizes of the D^miR-223^ HCT-116 cell spheres decreased within 72 h (t = −7.97, *p* < 0.001), while the sizes of WT^miR-223^ HCT-116 cell spheres remained unaffected (Figure 5B). LPS–ATP decreased the size of both D^miR-223^- and WT^miR-223^-expressing HCT-116 cell spheres at 72 h (Figure 5B, the differences between LPS–ATP-treated and untreated D^miR-223^ cells: t = −5.7; *p* = 0.005; between LPS–ATP-treated and untreated WT^miR-223^ cells: t = −6.7, *p* = 0.002). Additionally, single cells detached from the spheres and invaded the surrounding area in both treatments (Figure 5B). The sphere size of LPS–ATP-treated WT^miR-223^ HCT-116 cells was larger than that of LPS–ATP-treated D^miR-223^ HCT-116 cells at 72 h (t = 6.5; *p* = 0.003). However, while the mass of sphere-detached cell invasion increased around the untreated D^miR-223^-expressing HCT-116 spheres by 72 h (t = 3.6, *p* = 0.022), they invaded more slowly and less frequently around the untreated WT^miR-223^ compared to D^miR-223^-sexpressing spheres over 72 h (t = −3.6, *p* = 0.022, Figure 5B).

The size of D^miR-223^ HCT-15 cell spheres was not affected by time. However, WT^miR-223^ HCT-15 cell spheres decreased in size over 72 h (t = −2.48, *p* = 0.045, Figure S4B). Although LPS–ATP decreased the size of WT^miR-223^-expressing HCT-15 cell spheres (t = −3.40, *p* = 0.027), the decrease was negligible in LPS–ATP-treated D^miR-223^ HCT-15 cell spheres (Figure S4B). In addition, similar to HCT-116 spheres, the invasion of sphere-detached cells was lower in WT^miR-223^ HCT-15 spheres than in D^miR-223^ HCT-15 spheres (t = −17.5, *p* < 0.0001, Figure S4B).

LPS–ATP did not affect the size of D^miR-223^ spheres compared with that in untreated cells (Figure 5B and Figure S4B). However, the mass of sphere-detached cells around LPS–ATP-treated D^miR-223^ spheres was higher than that around untreated HCT-116 (: untreated: 3.481 ± 0.27 area pixel^2^, LPS–ATP: 5.114 ± 0.30 area pixel^2^, t = 6.6; *p* = 0.03; Figure 5B, HCT-15: untreated: 19.267 ± 0.63 area pixel^2^, LPS–ATP: 20.846 ± 0.61 area pixel^2^, t = 3.09; *p* = 0.036; Figure S4B). In contrast, LPS–ATP reduced the size of WT^miR-223^-expressing spheres compared to those of untreated over 72 h (HCT-116: t = −8.4, *p* = 0.001, Figure 5B; HCT-15: t = −2.1, *p* = 0.100, Figure S4B). However, the mass of detached cells invading the surrounding area LPS–ATP-treated WT^miR-223^spheres was larger than that of untreated spheres in 24 h (HCT-116: untreated: 2.485 ± 0.25 area pixel^2^, LPS–ATP: 3.501 ± 0.50 area pixel^2^, t = 7.3; *p* = 0.002; HCT-15: untreated: 10.070 ± 0.17 area pixel^2^, LPS–ATP: 13.162 ± 0.42 area pixel^2^, t = 11.6; *p* < 0.001). Also, LPS–ATP did not affect the number of detached cells and their migration further from the 48 h to the 72 h. These data suggest that miR-223 decelerates the loss of cell junctions on the outer surface of the HCT-116 and HCT-15 cell spheres (Figure 5B and Figure S4B).

### 2.6. miR-223 Recovered the Cytokines Production, Which Were Suppressed after NLRP3 Activation by LPS–ATP

**Figure 6:**
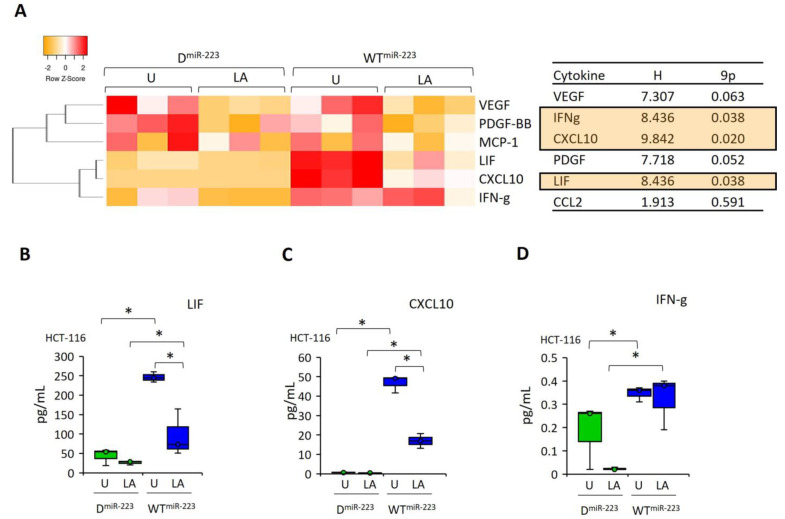
The effect miR-223 on cytokine secretion in inflammasome induced by LPS–ATP HCT-116 cells. (**A**)The heat map shows the changes in the levels of cytokine secretion patterns in HCT-116 cells after LPS–ATP and gli–LPS–ATP treatments. (**B–D**) The cytokines, which were affected by LPS–ATP. Data represent three biological replicates. The *p*-value was calculated using a Kruskal–Wallis test. H: Test statistic for the Kruskal–Wallis test. * *p* < 0.05 D: Decoy, WT: Wild type, U: Untreated, LA: LPS–ATP.

The secretion of CCL2, PDGF-BB, and VEGF was unaffected in both D^miR-223^- and WT^miR-223^-expressing HCT-116 cells (Figure 6A). In contrast, the release of LIF, CXCL10, and IFN-γ was higher in WT^miR-223^ cells than in D^miR-223^ cells (z = −1.96; *p* = 0.05, Figure 6B–D). Similarly, LIF, CXCL10, and IFN-γ secretion were higher in WT^miR-223^ than in D^miR-223^ cells after LPS–ATP treatment (z = −1.96, *p* = 0.05; Figure 6B–D). Notably, LPS–ATP decreased the secretion of LIF and CXCL10 in WT^miR-223^-expressing cells compared to untreated HCT-116 cells (z = −1.96; *p* = 0.05). Interestingly, these cytokines were unaffected in LPS–ATP-treated D^miR-223^ cells (Figure 6B,C). IFN-γ was not changed in LPS–ATP-treated WT^miR-223^ and D^miR-223^ cells compared to the untreated cells (Figure 6D).

## 3. Discussion

The role of NLRP3 is well-described in immunological cells, mainly macrophages, neutrophils, monocytes, and dendritic cells, in various immunological diseases, including cancer [30,31,32]. However, there are different opinions about the effect of NLRP3 on the cancerization process in cancer cells [33]. Because NLRP3 activation ultimately leads to cell death, it is thought that NLRP3 can be used as a treatment tool [34]. At the same time, opposing views argue that NLRP3-dependent death may increase tumor growth because it increases the release of proinflammatory cytokines [35].

In our previous studies, we showed that NLRP3 activation can be used as a treatment tool in cancer cells with low intrinsic NLRP3 activation capacity. However, increased NLRP3 levels in cancer cells with high NLRP3 activation capacity can increase tumor aggression [13]. Studies have shown that high NLRP3 expression advances CRC tumor characteristics and supports the microenvironment, facilitating metastasis [6,7,36]. Therefore, inhibiting NLRP3 has been suggested to suppress tumor aggression by reducing the release of inflammatory cytokines [5]. However, our knowledge about the effect of NLRP3 inhibitors on CRC cell progression remains incomplete.

Our previous study showed that VX765, an agent that blocks NLRP3-dependent cytokine release by suppressing caspase-1, increased cell proliferation in epithelial cancer cells, such as prostate, lung, and breast cancers [13]. Similarly, VX765 has been reported to have a protective effect on CRC cells [37,38]. These findings indicate that suppression of caspase-1 alone may not be sufficient for cancer cell inhibition. Therefore, in this study, NLRP3 activation was suppressed by blocking the P2X7 receptor, which is an upstream step of the NLRP3 signaling pathway, and by posttranscriptional suppression of NLRP3 translation. The effects of both forms of NLRP3 suppression on CRC cell progression were investigated.

The in vitro activation of NLRP3 requires external priming and activating stimuli in macrophages [39]. However, previous studies have shown that the priming signal of NLRP3 can be provided by tumor-produced IL-1 [40,41]. In addition, NLRP3 expression in cancer cells could differ depending on their intrinsic proinflammatory cytokines’ secretion capacity without the need for a priming stimulus, such as LPS [13]. To demonstrate the self NLRP3-activating capacity in CRC cells, this study analyzed the NLRP3 expression in CRC cells after ATP-only or LPS–ATP treatments. An increased NLRP3 expression was found after ATP-only compared to untreated cells, indicating the self-NLRP3 priming capacity of colon cancer cells. In addition, we demonstrated NLRP3 expression and gasdermin D cleavage after treatment with LPS only. This could be explained by the ability of LPS to activate NLRP3 without a second stimulus provided by a potassium efflux agent [13]. According to a previous study, an intrinsic stimulus, such as oncogene-induced reactive oxygen species, could serve as the second stimulus for NLRP3 activation in cancer cells [42]. However, as expected, the highest NLRP3 expression, as well as IL-1β and IL-18 secretion by CRC cells, occurred upon LPS–ATP stimuli. Therefore, LPS–ATP was applied to CRC cells for the remaining analysis.

LPS–ATP-induced NLRP3 activation decreased the proliferation of CRC cells. In line with this, NLRP3 activation decreased the size of spheres formed by CRC cells. However, cells on the outer surface of the spheres detached and invaded a larger area. Additionally, LPS–ATP increased the migration rate of CRC cell monolayers. These findings indicate that LPS–ATP-induced NLRP3 activation could lead to an inflammatory cell death that promotes metastasis [43]. This study found that inflammasome activation reduced the secretion of IFNg and CXCL10. The loss of IFN-γ secretion and IFN-γ inducible protein, CXCL10, induces the expression of cellular FLICE-like inhibitory protein (cFLIPL) and mixed lineage kinase domain-like (MLKL) [11,44]. cFLIPL and MLKL expression could promote necroptosis, leading to CRC recurrence [44,45]. In addition, IL-1β secretion promotes the invasiveness of CRC cells through the activation of EMT [44].

In contrast, the LPS–ATP decreased the secretion of platelet-derived growth factor-BB (PDGF-BB), CCL2, and VEGF in HCT-116 cells. PDGF-BB plays a role in tumor stroma development by inducing CCL2-dependent macrophage recruitment [46]. CCL2-recruited macrophages could promote TAM accumulation in the tumor microenvironment and the production of angiogenic factors, such as VEGF [46]. Exposure to extracellular matrix proteins, such as collagen, is required for PDGF-BB induction in the tumor microenvironment [47]. Considering that the experimental setup in this study lacks the components of a tumor microenvironment, the reduction in PDGF-BB, CCL2, and VEGF could be due to the absence of endothelium-exposing signals. Therefore, a better understanding of the effect of LPS–ATP on the function of PDGF-BB-CCL2 and VEGF may require further investigation in a CRC tumor-microenvironment model.

Several mechanisms control NLRP3 activation, including suppressing upstream signals, interrupting inflammasome assembly, blocking caspase-1 activation, and inhibiting gasdermin D cleavage [48]. These mechanisms could be applied as a therapeutic approach to treat CRC [5,12]. Our previous findings showed that VX765, a caspase-1 inhibitor, induces cancer cell proliferation and angiogenesis in neuroblastoma, glioblastoma, lung, prostate, and breast cancer cells [13]. In line with this, Chen et al. have demonstrated that VX765 promotes the proliferation of HT-29, a CRC cell line [38]. Another NLRP3 inhibitor, gli, was shown to suppress the formation of inflammasome assembly by inhibiting the P2X7 receptor, expressed in many cells within the tumor microenvironment [14]. The elevated expression of the P2X7 receptor was linked to increased migration and invasion in head and neck cancer and pancreatic ductal adenocarcinoma cell lines [49,50].

Gli, an inhibitor of the P2X7 receptor, has been reported to suppress proliferation and invasion by promoting apoptosis in breast, ovarian, prostate, gastric, liver, and bladder cancer cells [15,16,17,51]. This study found that, while gli was sufficient to suppress NLRP3 and its downstream products, it failed to inhibit the inflammasome-induced proliferation of HCT-116 and HCT-15 cells. Also, our previous study demonstrated that gli decreases LPS–ATP-mediated inflammatory cell death in cancer cell lines [13]. Previous research has suggested that gli promotes autophagy through the AMPK pathway [52]. In addition, gli-loaded engineered nanovectors can be beneficial as a tool to modulate the balance between inflammasome activation and autophagy in an in vitro model of Alzheimer’s disease-associated inflammation [53]. Also, in this study, gli induced RNA expression of ATG5 and BECN1 genes, which are involved in autophagy signaling [54].

Evidence has shown that autophagy could play either a pro-survival or pro-death role in cancer prognosis [55]. Autophagy-induced cancer cell survival contributes to developing drug resistance, self-renewal, and CSC maintenance [55,56]. In this study, the invasion area of sphere-detached cells was larger in gli–LPS–ATP-treated spheres than in LPS–ATP-treated spheres. Additionally, the enlargement of the spheres of gli–LPS–ATP-treated cells could suggest that tumor self-renewal by activating multipotent CRC cells in the presence of inflammatory stimuli [57]. Although multiple studies have reported the antiproliferative and proapoptotic effects of gli in cancer cells in vitro [17,58,59], epidemiological evidence has associated the use of gli with an increased mortality rate in various malignancies, including pancreatic and lung cancer [60,61,62]. The findings of this study confirm that caution should be exercised regarding the use of gli in CRC patients with high NLRP3 because it could promote invasion and metastasis.

The posttranscriptional regulatory function of miRNAs on mRNA stability and translation represents an alternative approach to inhibiting NLRP3 production [63]. According to the Target Scan Human database, the 3′UTR of NLRP3 has a conserved binding site for miR-223 (Figure 4A). Studies have demonstrated that miR-223 transcriptional suppression of NLRP3 could promote apoptosis in breast cancer, hepatocellular cancer, cervical cancer, and glioblastoma cells [64,65,66,67]. In contrast, elevated miR-223 expression was associated with advanced tumor stage in oral squamous carcinoma [68]. In this study, miR-223 overexpression reduced NLRP3 expression, cleavage of gasdermin D, and the secretion of proinflammatory cytokines IL-1β and IL-18 in HCT-116 cells. In addition, although miR-223 increased the expression of autophagy-involved genes ATG5 and BECN1, it also induced proapoptotic Bax expression. BAX has been shown to induce mitochondrial autophagy through selective removal of mitochondria, which also leads to cytochrome release and enhanced apoptosis [69]. In addition, the decreased migratory capacity of CRC cells expressing miR-223 could be explained by increased secretion of IFN-γ [70] and CXCL10 [71,72]. IFN-γ could reduce migration by promoting cell apoptosis through JAK-STAT1-caspase signaling [73,74]. In addition, CXCL10 could be involved in the anti-migratory and apoptosis-promoting signaling pathways by activating the chemokine receptor CXCR3-B [71,72].

In this study, during sphere formation, cells expressing decoy miR-223 had reduced sphere size, while miR-223-expressing cells showed no change in sphere size over a 72 h time period. However, despite the decreased invasion from the spheres, the invasiveness in miR-223-expressing cells was not completely eliminated. This could be attributed to the dual role of miR-223-induced cytokines. While IFN-γ-induced JAK-STAT1 signaling promotes apoptosis, it also mediates the upregulation of EMT transcription factors by inducing IFN-γ-induced protein with tetratricopeptide repeats 5 (IFIT5) [75]. Also, studies have shown that IFN-γ transforms cancer stem cells into a metastatic form by inducing CXCR4 production and contributing to the microenvironment for cell invasion [76]. Similarly, while the binding of CXCL10 to the chemokine receptor CXCR3-B inhibits cancer cell proliferation, it can also activate another splice variant of CXCR3, CXCR3-A, which can induce chemotaxis and cancer cell proliferation [71,77]. In addition, while LIF expression, which was found to be increased in miR-223-expressing cells, reduces the stem-like cell phenotype [33], it conversely induces JAK/STAT3 activation to promote migratory and metastatic features of the CRC cells [78,79]. Therefore, the effect of miR-223-mediated NLRP3 inhibition on CRC cells could be influenced by the different functions of IFN-γ, CXCL10, and LIF in the cell.

## 4. Methods

### 4.1. Cell Lines and Reagents

HCT-116, the human colorectal carcinoma cell line, was obtained from the American Type Culture Collection (ATCC; Rockville, MD, USA). In addition, HCT-15, the human colon adenocarcinoma cell line, was a gift from Dr. Bondar, Kazan Federal University. Cells (passage 15) were grown in Dulbecco’s Modified Eagle’s Medium-F12 (DMEM/F12; PanEco, Moscow, Russia), supplemented with 10% fetal bovine serum (HyClone, Logan, UT, USA), 50 U/mL penicillin, 50 µg/mL streptomycin (PanEco, Moscow, Russia), 2 mM L-glutamine (Capricorn Scientific, Darmstadt, Germany), and 1 mM sodium pyruvate (PanEco, Moscow, Russia) in a 5% CO_2_ humidified incubator at 37 °C. Cell lines were used in passage 15 in all experiments.

Lipopolysaccharide (LPS) from Escherichia coli O111:B4 (L4391), ATP (A3377), and Glibenclamide (gli) (G0639) were obtained from Sigma (St. Louis, MO, USA).

### 4.2. Plasmid Transfection

The low sensor backbone (LSB)-hsa-miR-223-3p (cat no: 103369; Addgene, Cambridge, MA, USA) plasmid was constructed to transfect HCT-116 cells using lipofectamine 3000 (ThermoFisher Scientific, Waltham, MA, USA) to sense miR-223 activity following the manufacturer’s instructions. The miR-223 activity was measured through the repression of mKate2 fluorescent protein, which is regulated by the target sites in the 3′ UTR of miR-223, relative to the transfection marker EBFP2 fluorescent protein [80]. In addition, a lentivirus containing decoy miR-223 was used as a negative control. The lentivirus was generated by transient cotransfection of HEK293T cells with three plasmids: shell (pCMV-VSVG), packing (psPAX2), and vector (decoy AB.pCCL.sin.cPPT.U6.miR-223-Decoy.hPGK.GFP.WPRE, cat no: 46601, Addgene, Cambridge, MA, USA), according to manufacturer’s instructions [81]. Lentivirus-containing supernatant was harvested after 36, 48, and 72 h posttransfection. Confocal microscopy was employed to visualize transfection efficiency.

### 4.3. Western Blot

Total protein was extracted using radioimmunoprecipitation assay buffer (Pierce BCA Protein Assay Kit; Thermo Fischer Scientific, Inc., Waltham, MA, USA), separated by gel electrophoresis (8–12% two-gradient polyacrylamide gel) and immune-blotted onto PVDF membranes (Biorad, Hercules, CA, USA). Membranes were blocked with 5% nonfat milk for 1 h and incubated with rabbit anti-NLRP3 (1:1000, Abcam, Cambridge, MA, USA), rabbit anti-GSDM (1:500, Sigma, St. Louis, MO, USA), or rabbit Anti-BAX (1:1000, Abcam, Cambridge, MA, USA) primary antibodies at 4 °C overnight. The membranes were then washed three times with PBS containing 0.1% Tween 20 and incubated with the secondary antibody, anti-rabbit IgG (1:2000, Santa Cruz Biotechnology, Heidelberg, Germany), for 2 h at room temperature (RT). A mouse monoclonal anti-actin beta antibody conjugated to HRP (1:2000, Sigma, St. Louis, MO, USA) was used to normalize protein expression for total protein. Clarity Western ECL Substrate (Bio-Rad, Hercules, CA, USA) revealed the primary–secondary antibody reaction, and ChemiDoc XRS Plus (Bio-Rad, Hercules, CA, USA) detected the protein band signals. ImageJ 1.53s software (NIH, Bethesda, MD, USA) was utilized to quantify the signal intensity.

### 4.4. Enzyme-Linked Immunosorbent Assay (ELISA)

IL-1β and IL-18 were analyzed using an ELISA kit according to the manufacturer’s recommendations (Vector-Best, Novosibirsk, Russia). Briefly, 100 μL of IL-1β and IL-18 standards or cell-free medium of samples were added to human anti-IL-1β and anti-IL-18 antibody-precoated microwells. The microtiter plate was then incubated for two hours at room temperature (RT) and washed with PBS containing 0.5% Tween 20. Afterward, biotinylated anti-IL-1β-, anti-IL-18 antibodies, and horseradish peroxidase (HRP)–streptavidin were added to the wells for 1 h at RT. In the last step, a substrate solution was added in the dark at RT for 30 min, and the enzymatic reaction was stopped using a stop solution provided with the kit. The absorbances of the immune complexes formed in the wells were read using a TECAN Infinite 200 microplate reader (Grödig, Austria) at OD450 nm with a reference reading at OD650 nm. Data were calculated according to the standard curves generated by the reference standards of IL-1β and IL-18 and reported as an average of three technical repeats.

### 4.5. Real-Time qPCR

Total RNA was extracted using TRIzol (Sigma, St. Louis, MO, USA), as described previously [82]. The concentration and purity (A260/280 ratio and A260/230 ratio) of the extracted RNA were assessed using a Thermo Scientific NanoDrop™ 1000 Spectrophotometer (Thermo Scientific, Waltham, MA, USA). A total of 10 ng of total RNA was used to synthesize cDNA with the RevertAid First Strand cDNA Synthesis Kit (Thermo Fisher Scientific, Inc., Waltham, MA, USA) for gene expressions and the miRCURY^®^ LNA^®^ RT Kit (Qiagen, Hilden, Germany) for miRNA expression analyses. The expression of NLRP3 (NLRP3) was analyzed using primers as described by Tezcan et al. [83]. The primers for Beclin 1 (BECN1), and ATG5 were designed using the GenSmart™ Design Tool (GenScript, Piscataway, NJ, USA) (Table 1). RNA input was normalized using a housekeeping gene, Actin β (ACTB), for gene expression. For miRNA expression analysis, qPCR was performed in a 10 µL reaction mixture (3 μL (60× diluted) of cDNA, 1 μL miRCURY LNA miRNA PCR Assay, (YP00205986, Qiagen, Hilden, Germany), 5 μL 2× miRCURY SYBR Green Master Mix (Qiagen, Hilden, Germany)) and 1 μL RNase-free water. The cycle parameters were as follows: 95 °C for 2 min and 40 cycles at 95 °C for 10 s and 56 °C for 60 s, in the CFX384 Touch™ Real-Time PCR Detection System (Biorad, Hercules, CA, USA). Copy numbers in the sample and the Ct value for miRNA expression were determined using the CFX384 Touch™ Real-Time PCR Detection System software (Biorad, Hercules, CA, USA). The 2^−ΔCt^ method was used to calculate the fold change in gene expression.

### 4.6. MTS Assay

Cell proliferation was assayed using the MTS test. Briefly, 2 × 10^3^ cells were seeded into each well of a 96-well plate. After treatment, 100 μL of cell-continued medium was collected, mixed with 20 μL MTS reagent (Sigma, St. Louis, MO, USA), and incubated at 37 °C for 2 h. Absorbance was measured at 490 nm using a TECAN Infinite 200 microplate reader (Grödig, Austria).

### 4.7. Annexin V assay

Cell viability was assessed using the APC Annexin V Apoptosis Detection Kit (Sony Biotechnology, San Jose, CA, USA). Briefly, cells were harvested in an Annexin V binding buffer and stained with the annexin labeling solution, which consisted of 2% annexin-V–APC and 0.1 µg/mL propidium iodide (PI), in the dark for 15 min. The cells were plotted using a BD FACSAria III Flow Cytometer (BD Biosciences, San Diego, CA, USA), and the FlowJo software package (FlowJo LLC, Ashland, OR, USA) was used to process the data. Cells positive for annexin-V-APC-only were counted as early apoptotic. In contrast, cells positive for Annexin V and PI or PI-only were considered late-apoptotic or non-apoptotic, respectively [84].

### 4.8. Scratch Wound Healing Assay

A confluent monolayer of cells was scratched by dragging a 200 μL pipette tip, and the debris was removed by washing with PBS. NLRP3 activation was induced using LPS/ATP or suppressed through preincubation with gli. Microscopic images of the wounded area were taken immediately after the scratch and 24 h after ATP treatment using an Axiovision Rel 4.5 software with a Zeiss AxioObserver.Z1 microscope (Göttingen, Germany). Changes in the wound size were measured using the same software. Each experiment was performed with three technical replicates.

### 4.9. Sphere-Invasion Assay

A number of 96-well U-bottom plates were coated with 2.5% Matrigel Basement Membrane Matrix to create a low attached surface. Cells (0.5 × 10^4^/well) were seeded onto each well of the microplate and centrifugated at 560× *g* for 10 min. After 48 h of incubation (5% CO_2_, 37 °C), the cells began to form spheroids and were then treated with LPS and ATP. Sphere size and counts were obtained before LPS (1 µg/mL) (time 0) and ATP (5 mM) treatments (30 min and 24–48 h) with a Zeiss Observer Z1 inverted microscope using Axiovision Rel 4.5 software (Göttingen, Germany).

### 4.10. Multiplex Analyzes

The Bio-Plex Pro™ Human Cytokine Screening Panel (48 plex) was used to analyze the cell-secreted cytokines following the manufacturer’s directions (Bio-Rad Laboratories, Hercules, CA, USA). Briefly, a cell-free medium (50 µL) was used to determine the cytokine concentrations. Data were collected using a Luminex 200 analyzer with a MAGPIX analyzer (Luminex, Austin, TX, USA) and MasterPlex CT control and analyzed using MasterPlex QT analysis software (MiraiBio, San Bruno, CA, USA). Each sample was analyzed in triplicate. The average cytokine levels were presented as a heatmap using the web-based program Heatmapper (http://www.heatmapper.ca/ (accessed on 11 November 2023) [85].

### 4.11. Statistical Analysis

Statistical analysis was performed using IBM SPSS Statistics for Windows (version 20) software (IBM Corp., Armonk, NY, USA). One-way ANOVA with Tukey’s post hoc analysis was utilized to evaluate the findings of the MTS assay, Annexin V, western blot and ELISA. An independent sample *t*-test was used to evaluate wound-healing and sphere formation assays and RNA expressions. The Kruskal–Wallis test for comparisons between individual experimental groups was performed for multiplex cytokine analyses. Data are presented as mean ± standard error. Significance was established at a value of *p* < 0.05.

## 5. Conclusions

The findings of this study highlight that NLRP3 induction leads to an immediate inflammatory death of CRC cells, possibly leading to a worsened outcome due to the proinflammatory cytokine secretion and metastasis promotion. However, because inhibiting NLRP3 with gli fails to suppress cancer cell growth and the metastatic phenotype, there may be drawbacks to its use in CRC. It is important to note that our study was conducted using only HCT-116 and HCT-15 cells. HCT-116 cells were collected from the colon and represent a known mutation [86]. In addition, HCT-15 cells are Dukes-type C-adenocarcinomas collected from the large intestine [86]. Therefore, the effects of gli and miR-223 observed in our study may not be applicable to other types of CRC, such as mucinous adenocarcinoma or genetically inherited carcinomas. Nevertheless, our data suggest that suppressing NLRP3 translation by targeting the 3′UTR region with miR-223 could be more effective than using gli to silence NLRP3 in CRC adenocarcinoma. This conclusion is based on the finding that miR-223 could promote apoptosis in HCT-116 and HCT-15 cells with more success than gli. However, the sole use of miR-223-mediated NLRP3 suppression may not be sufficient to prevent CRC metastasis.

## Figures and Tables

**Table 1:** The primers used in RT-qPCR.

ACTB	F	5′-GAC AGG ATG CAG AAG GAG ATT ACT-3	[30]
	R	5′-TGA TCC ACA TCT GCT GGA AGG T-3′	
NLRP3	F	5′-ATG AGT GCT GCT TCG ACA TC-3′	[30]
	R	5′-TTG TCA CTC AGG TCC AGC TC-3	
BECN1	F	5′-AGA CCC AGG AGG AAG AGA CT-3′	
	R	5′-AGC TGT TGG CAC TTT CTG TG-3′	
ATG5	F	5′-CTG GGC TGG TCT TAC TTT GC-3′	
	R	5′-GGC CAA AGG TTT CAG CTT CA-3′	

## Data Availability

Data is contained within the article and Supplementary Materials.

## Supplementary Materials

The following supporting information can be downloaded at https://www.mdpi.com/article/10.3390/ph17030299/s1. Figure S1: Gli-mediated NLRP3 suppression in HCT-116 and HCT-15 cells, Figure S2: The effect of gli-inhibition of NLRP3 on migration and sphere formation of HCT-15 cells, Figure S3: miR-223 mediated suppression of NLRP3 in HCT-116 and HCT-15 cells, Figure S4. The effect of miR-223 on migration and sphere formation of HCT-15 cells.
