# Supplementary material for: Implications of NLRP3 Suppression Using Glibenclamide and miR-223 against Colorectal Cancer
Source: Pharmaceuticals (Basel). 2024 Feb 26;17(3):299. doi: 10.3390/ph17030299 (PMC10975274; doi:10.3390/ph17030299)
Supplement: Supplementary file 1 [file pharmaceuticals-17-00299-s001.zip › pharmaceuticals-2826623-supplementary.pdf]

Implication of NLRP3 Suppression by Glibenclamide and miR-223 Against Colorectal Cancer

Supplementary Figures

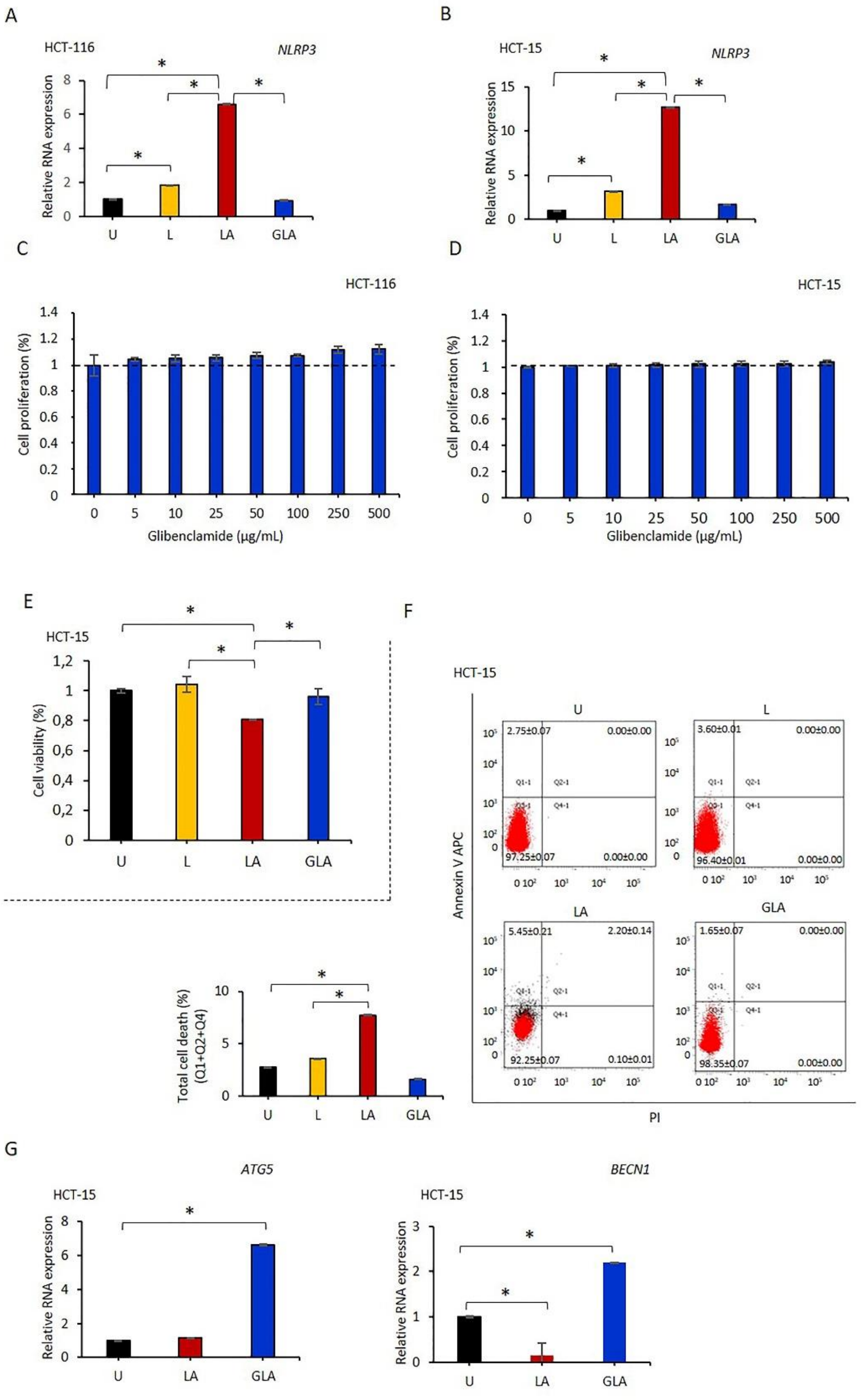

Figure S1. Gli-mediated NLRP3 suppression in HCT-116 and HCT-15 cells. (A) RNA expression of NLRP3 after LPS-only, LPS+ATP and Gli+LPS+ATP in HCT-116 and (B) HCT-15 cells. (C) A dose dependent effect of Gli on HCT-116 and (D) HCT-15 cell viability in MTS assay. (E) The effect of LPS-only, LPS+ATP and Gli+LPS+ATP on HCT-15 cell proliferation. The MTS assay values of treated cells were normalized to the untreated sample. (F) The Annexin V assay of HCT-15 cell viability. (F) ATG5 and BECN1 RNA expression levels of HCT-15 cells. Data represent three biological replicates P-value was calculated using the one-way ANOVA model with Tukey's post hoc tests. * $p < 0.05$. U: Untreated, L: LPS only, LA: LPS-ATP, GLA: Gli-LPS-ATP

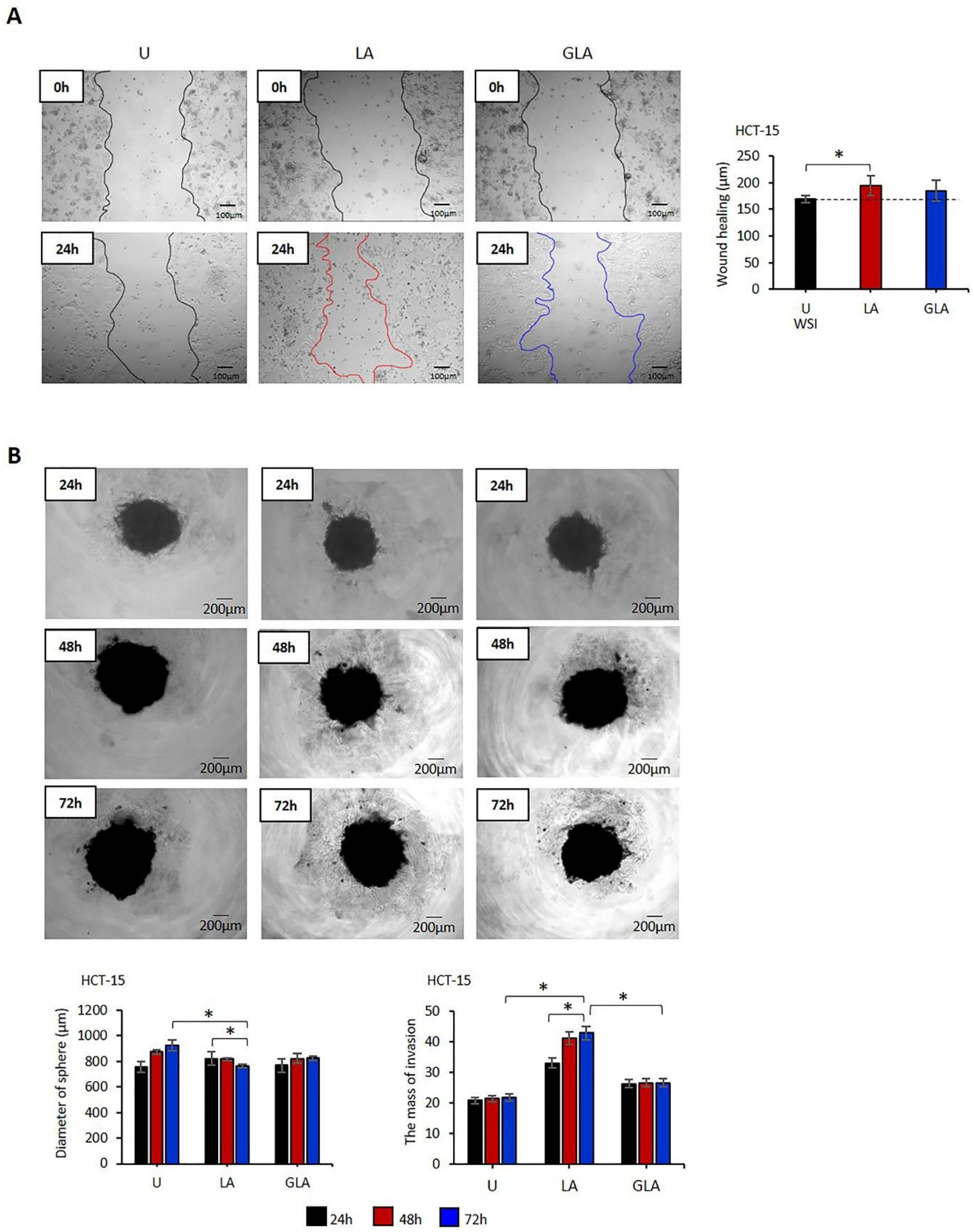

Figure S2. The effect of gli-inhibition of NLRP3 on migration and sphere formation of HCT-15 cells. (A) Wound-healing of HCT-15 cells. Images were captured before LPS treatment (time 0) and 24h after ATP treatment. (B) Sphere formation and invading capacity of HCT-15 cells. Images were captured 24–72 h after ATP treatment. Images were analyzed using Image J 1.53s software (NIH, USA). Data represent three biological replicates. The P-value was calculated using an independent sample T-test. * $p<0.05$ U: Untreated, LA: LPS-ATP, GLA: Gli-LPS-ATP, WSI: Wound size at the initiation.

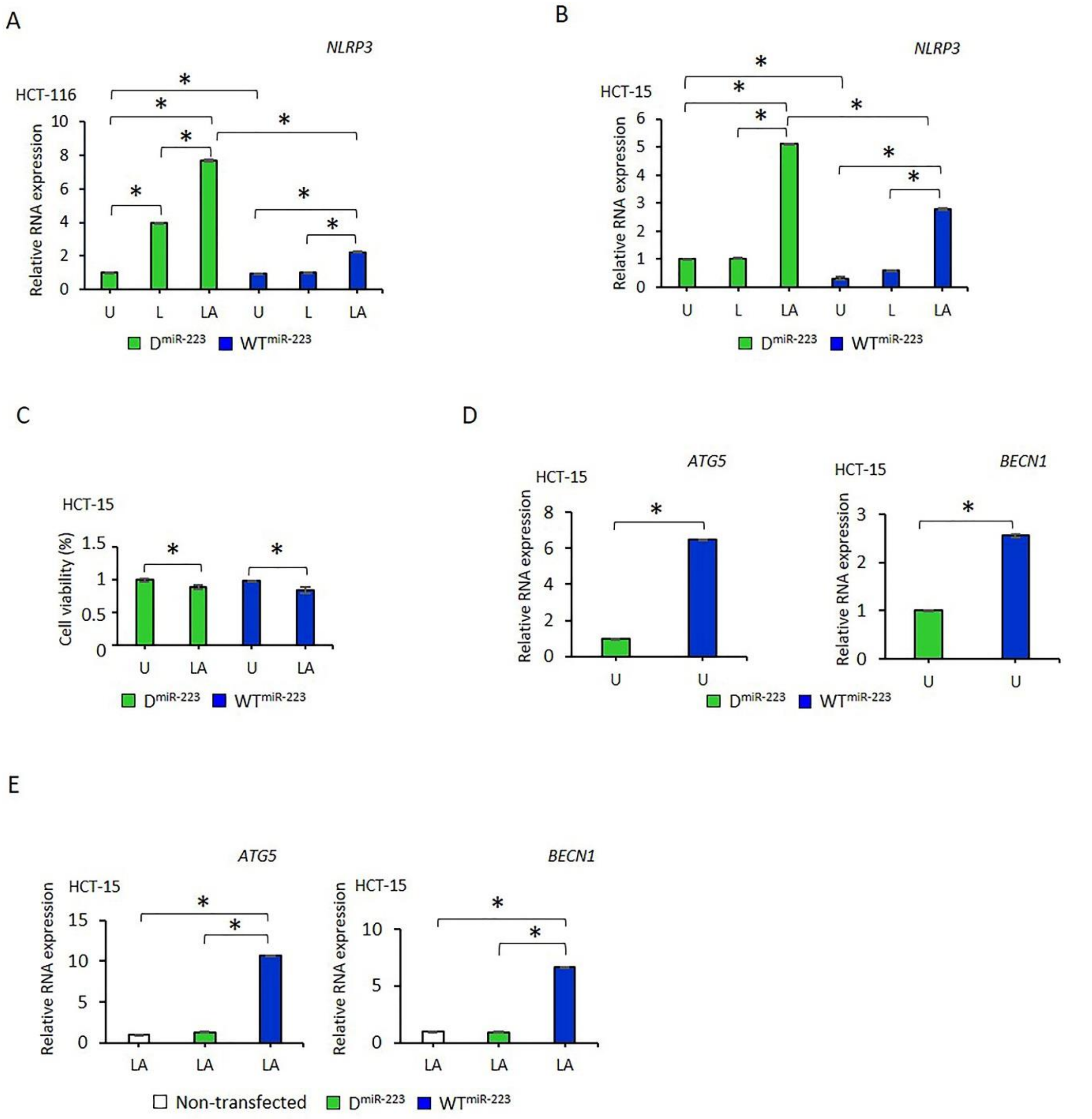

Figure S3. miR-223 mediated suppression of NLRP3 in HCT-116 and HCT-15 cells. (A) RNA expression of NLRP3 in $WT^{miR-223}$ and $D^{miR-223}$ expressing HCT-116 and (B) HCT-15 cells. (C) The cell proliferation of $WT^{miR-223}$ and $D^{miR-223}$ expressing HCT-15 cells in MTS assay. (D) ATG5 and BECN1 RNA expression levels in untreated and € LPS+ATP treated $WT^{miR-223}$ and $D^{miR-223}$ expressing HCT-15 cells. P-value was calculated using the one-way ANOVA model with Tukey's post hoc tests. * $p < 0.05$. WT: wild type, D: Decoy, U: Untreated, LA: LPS-ATP.

A

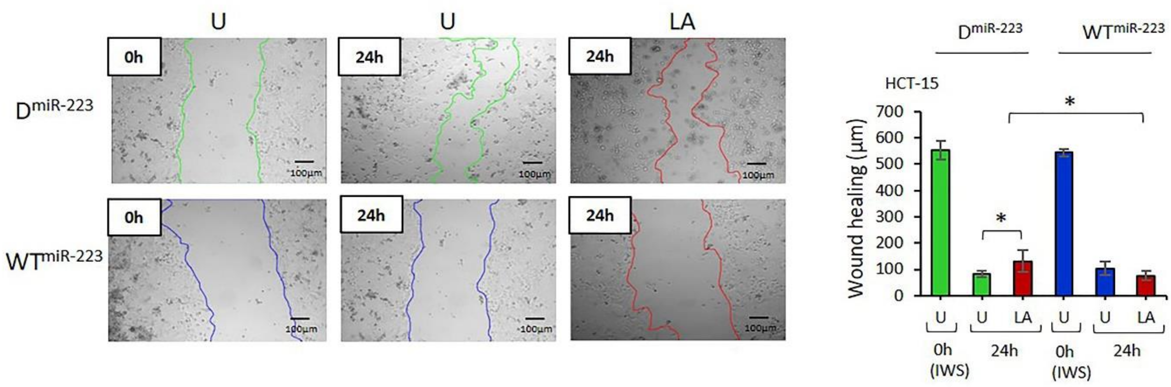

B

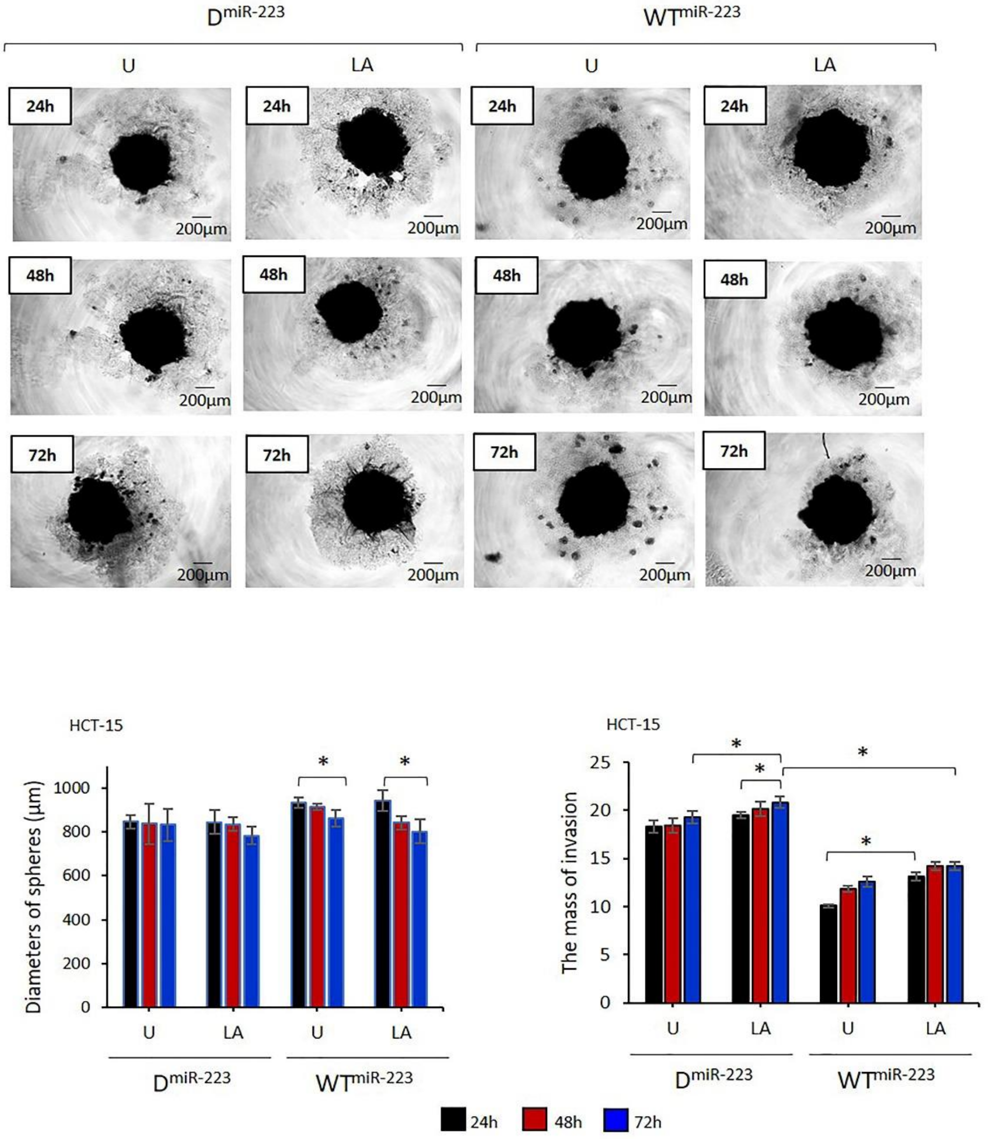

Figure S4. The effect of miR-223 on migration and sphere formation of HCT-15 cells. (A) Wound-healing of HCT-15 cells. Images were captured before LPS treatment (time 0) and 24h after ATP treatment. (B) Sphere forming and invading capacity of HCT-15 cells. Images were captured 24–72 h after ATP treatment. Images were analyzed using Image J 1.53s software (NIH, USA). Data represent three biological replicates. p-value was calculated using an independent sample T-test. * $p<0.05$ D: Decoy, WT: Wild type, U: Untreated, LA: LPS-ATP, WSI: Wound size at the initiation.
